# A longitudinal study of non-medical determinants of adherence to R-CHOP therapy for diffuse large B-cell lymphoma: implication for survival

**DOI:** 10.1186/s12885-015-1287-9

**Published:** 2015-04-15

**Authors:** Cécile Borel, Sébastien Lamy, Gisèle Compaci, Christian Récher, Pauline Jeanneau, Jean Claude Nogaro, Eric Bauvin, Fabien Despas, Cyrille Delpierre, Guy Laurent

**Affiliations:** 1 Department of Haematology, Toulouse University Hospital, Toulouse, France; 2 University of Toulouse III Paul Sabatier, Toulouse, France; 3 INSERM UMR1027 (The French National Institute of Health and Medical Research), Toulouse, France; 4 Department of Clinical Pharmacology, Toulouse University Hospital, Toulouse, France; 5 Health care cancer network Oncomip, Toulouse, France; 6 INSERM UMR1037 (The French National Institute of Health and Medical Research), Cancer Research Centre of Toulouse, Toulouse, France

**Keywords:** Treatment adherence, Relative dose-intensity, Lymphoma, Non-medical determinant of health, Overall survival

## Abstract

**Background:**

Adherence to therapy has been established for years as a critical parameter for clinical benefit in medical oncology. This study aimed to assess, in the current practice, the influence of the socio-demographical characteristics and the place of treatment on treatment adherence and overall survival among diffuse large B-cell lymphoma patients.

**Methods:**

We analysed data from 380 patients enrolled in a French multi-centre regional cohort, with diffuse large B-cell lymphoma receiving first-line treatment with R-CHOP (rituximab, cyclophosphamide, doxorubicin, vincristine, prednisone) or R-CHOP-like regimens. Direct examination of administrative and medical records yielded the date of death. We studied the influence of patients’ socio-demographic characteristics and place of treatment on the treatment adherence and overall survival, adjusted for baseline clinical characteristics. Treatment adherence was measured by the ratio between received and planned dose Intensity (DI), called relative DI (RDI) categorized in “lesser than 85%” and “at least 85%”.

**Results:**

During the follow-up, among the final sample 70 patients had RDI lesser than 85% and 94 deceased. Multivariate models showed that advanced age, poor international prognosis index (IPI) and treatment with R-CHOP 14 favoured RDI lesser than 85%. The treatment in a public academic centre favoured RDI greater than or equal to 85%. Poor adherence to treatment was strongly associated with poor overall survival whereas being treated in private centres was linked to better overall survival, after adjusting for confounders. No socioeconomic gradient was found on both adherence to treatment and overall survival.

**Conclusions:**

These results reinforce adherence to treatment as a critical parameter for clinical benefit among diffuse large B-cell lymphoma patients under R-CHOP. The place of treatment, but not the socioeconomic status of these patients, impacted both RDI and overall survival

## Background

Diffuse large B-cell lymphoma (DLBCL) is one of the most frequent histological subtypes among Non-Hodgkin’s lymphomas (NHL) [1]. DLBCL course is naturally aggressive due to rapid tumour progression, visceral propagation, and metabolic complications related to lysis syndrome. However, DLBCL is a chemosensitive disease for which anthracyclin-based chemotherapy with CHOP was found to be effective since its introduction in the late seventies [2]. During the last decade, chemotherapy further improved through the development of immunochemotherapy consisting in the addition of rituximab (R) to CHOP (rituximab, cyclophosphamide, doxorubicin, vincristine, prednisone) or CHOP-derived regimens [3,4]. R-CHOP administered each 21 days (R-CHOP21) has become the standard for front-line treatment for DLBCL based on the pivotal LNH-98-5 study of the Grouped’ Etude des Lymphomes de l’Adulte (GELA) [3]. However, some variants of treatment have been designed in order to increase CHOP intensity by shortening the intercourse period, such as the R-CHOP14 protocol (given each 14 days) promoted by the German Lymphoma Study Group, and/or by increasing doses such as the R-ACVBP (rituximab, doxorubicin, cyclophosphamide, vindesine, bleomycin and prednisone) protocol derived from the GELA studies. In the GELA network, despite its higher toxicity compared to CHOP, R-ACVBP has become the standard for young patients with high international prognosis index (IPI) scores [5]. Finally, low-intensity chemotherapy, such as R-mini-CHOP, has been developed in elderly patients with age older than 80 years and was found to be tolerable and reasonably effective in this context [6].

In spite of adaptation to age and supportive care, including widespread use of hematopoietic growth factors (HGF), R-CHOP and R-CHOP derived protocols induce significant toxicities with life-threatening complications, like febrile neutropenia, sepsis and severe gastro-intestinal toxicities. Treatment-related mortality (TRM) remains relatively low in younger patients (2-5%) but could reach up to 8% for patients older than 60 years-old [3,7,8]. Intolerance to treatment often results in reducing treatment intensity, and consequently, non-adherence to the treatment protocol. Adherence to a chemotherapy regimen can be measured either by the ratio between the number of cycles administered and planned, or by the relative dose-intensity (RDI) which is the amount of drug delivered per time unit, compared to doses defined in the treatment protocol [9]. Dose concession is considered as a key issue in the treatment of patients with DLBCL [9-14].

The influence of RDI on outcome in CHOP therapy was first described by Epelbaum and co-workers more than 20 years ago with significant higher response rates for DLCBL patients who presented a better adherence to treatment [15]. Following this pioneer study, several reports have confirmed that higher RDI correlated with prolonged survival among NHL [11], including DLBCL [9,10,15,16]. Other studies found that poor treatment adherence assessed by the RDI was, besides age and IPI, one of the most potent predictors for survival [10,12]. The introduction of rituximab at the end of the 90s has reopened this question as two studies showed that, in DLBCL treated with R-CHOP, treatment adherence correlated with prolonged survival in multivariate analysis with several cut-offs of RDI [13,14]. Factors predicting RDI have been already identified in at least five cohort studies listed in a recent review. The most significant predictors were age older than 60–65 years, followed by of the Eastern Cooperative Oncology Group performance (ECOG) status, type of RCHOP therapy (ACVBP versus standard CHOP), IPI and use of G-CSF [17].

Besides such parameters related to patient physical characteristics or to the disease, the socioeconomic status (SES) and the place of treatment might also interfere with RDI. Indeed, some socioeconomic characteristics, as the level of education and the occupational status, have already been shown to be associated with treatment access and survival among patient with NHL [18-21]. Although these disparities could not be entirely related to chemotherapy administration, they may reflect differences in healthcare quality level and therefore, raise the possibility that the administration of chemotherapy can be also affected. Alternatively, since it has been shown that the place of treatment (academic versus community centre) may also influence overall survival of DLBCL patients [22], it could also be possible that this parameter influences the RDI.

In this study, we investigate the adherence to chemotherapy in current practice in a French health care system in a prospective cohort of patients treated for DLBCL with R-CHOP or R-CHOP derived regimens. More specifically, we study treatment adherence determinants by distinguishing the clinical characteristics, the socio-demographical characteristics of patients including their socioeconomic status, the place of treatment. Finally, we study the association between RDI and mortality.

## Methods

### Study design and population

This work is based on data from an ongoing prospective cohort of DLBCL patients in the French Midi-Pyrénées region, in the southwest of the country: the AMARE cohort. Patients were included if they received first-line treatment for DLBCL with R-CHOP or R-CHOP-like regimens from November 2006 without age restriction, in the main centres covered by the regional cancer network. Patients were excluded if they displayed central nervous system involvement, HIV infection, solid organ transplantation or previous documented indolent NHL. All patients signed informed consent before inclusion in this network. The study was approved by the local ethical committee of the Toulouse University Hospital.

### Data collection

Data were collected by one person through direct examination of administrative and medical records of the 418 patients treated between November 2006 and June 2011 (last follow up in June 2014). During the follow-up, information was gathered regarding treatment-related events and vital status, including the date of the events.

### Socio-demographical characteristics of patients

Patients’ characteristics included severe comorbidity (none; at least one among chronic or viral hepatitis, cardiovascular or metabolic disease, autoimmune disease or cancer) and social characteristics at diagnosis. The last one encompassed occupational status (active; inactive) and marital status (alone; not alone) at diagnosis. In addition, we used the European ecological deprivation index (EDI) built from patients’ addresses as a proxy of their individual socioeconomic status [23]. The geographical units used were IRIS as defined by the National Institute for Statistics and Economic Studies (INSEE), whereby an IRIS represented the smallest geographical census unit available in France, including approximately 2000 individuals with relatively homogeneous social characteristics. The regional capital and other major towns are divided into several IRIS and small towns form one IRIS. A score of social deprivation has been attributed to each IRIS: the higher the score, the higher the level of social deprivation. We used quintile of social deprivation as a proxy of the individual socioeconomic status, the highest quintile corresponding to the lowest socioeconomic status [23].

### Clinical characteristics

At diagnosis were collected: age (coded in tertile in our models), gender, the presence or absence of systemic (B) symptoms; the Ann Arbor stage (localized (Ann Arbor stage I or II) or advanced (Ann Arbor stage III or IV); the serum Lactate Dehydrogenase (LDH) concentration (normal or elevated); the ECOG performance status (PS) (PS = 0 or 1 (good); PS = 2, 3, or 4 (poor)) [24] and the IPI [25,26]. As it already accounted for each of the three former prognosis factors completed by the presence of more than one extra nodal site and age older than 60 years-old, the IPI score was used in our analyses in order to limit the number of variables to adjust for in statistical models and coded in three prognostic groups as suggested by Sehn et al. for DLBCL patients treated with R-CHOP: very good for IPI = 0, good for IPI =1 or 2 and poor for IPI = 3, 4 or 5 [27]. Regimens have already been described elsewhere [3,5,8,28]. Treatment followed the GELA recommendations or trials relevant to this period. Supportive care consisted of valacyclovir, sulfamethoxazole-trimethoprim and granulocyte colony-stimulating factor (G-CSF) primary prophylaxis for all.

### Place of treatment

The treatment centres encompassed six public centres (1 academic and 5 non-academic hospitals) and three private centres which were categorized as private centres, public academic centres (Toulouse University Medical Centres (TUMC)), or public community hospitals.

### Adherence to treatment

For each patient, adherence to treatment was assessed using the ratio between received and planned dose intensity as described by Epelbaum et al. [9]. For each patient, dose intensity (DI) was calculated, by direct examination of pharmacist records and by dividing the total actual dose of each drug by the time needed to deliver it. The expression of the actual DI as a fraction of the stated dose was defined as relative DI (RDI). In this study, we calculated RDI for the principal drugs, i.e. cyclophosphamide and doxorubicin. As the classification of patients between the groups “poor adherence” and “good adherence” was similar for the two drugs, only those for doxorubicin are shown. In the RDI calculation, we considered the following planned dose intensities for doxorubicin: 8 cycles of 21 days with 50 mg/m^2^ for R-CHOP21 and R-CHVP (rituximab, cyclophosphamide, doxorubicin, etoposide, prednisone), 8 cycles of 14 days with 50 mg/m^2^ for R-CHOP14, 8 cycles of 21 days with 25 mg/m^2^ for R-miniCHOP and R-miniCHVP, 4 cycles of 14 days with 75 mg/m^2^ for R-ACVBP. We used a cut-off value reduction of 15%, based on the study of Lyman et al. [29].

### Survival

Overall Survival (OS) was calculated from the first day of the first chemotherapy until death of any cause. These data were found in the medical records during the follow-up visits at the centres followed in the study.

### Statistical analysis

Patients included in the cohort were described by quintile of social deprivation index to give an overview of the social distribution of the characteristics related to the disease, the patient and care modalities. Then, we built multivariate models for analyzing RDI (RDI < 85% or ≥85%) and survival including all variables associated with these outcomes in bivariate analyses at the threshold of 0.2 (data not shown). A logistic regression model was performed to identify determinants of RDI. Regarding survival analyses, Kaplan-Meier survival curves were plotted and compared using the log-rank test. Then a Cox model was performed to identify determinants of survival, including RDI. For all models, conditions of application and models fit were checked by using Hosmer and Lemeshow for the logistic model and by analysing Schoenfeld residuals for the Cox model. As the proportional hazard assumption was violated for treatment adherence, we used a Cox model with time-varying coefficient. All the analyses were done by using STATA release 12 (StataCorp LP, College Station, TX, USA).

## Results

**Figure 1:**
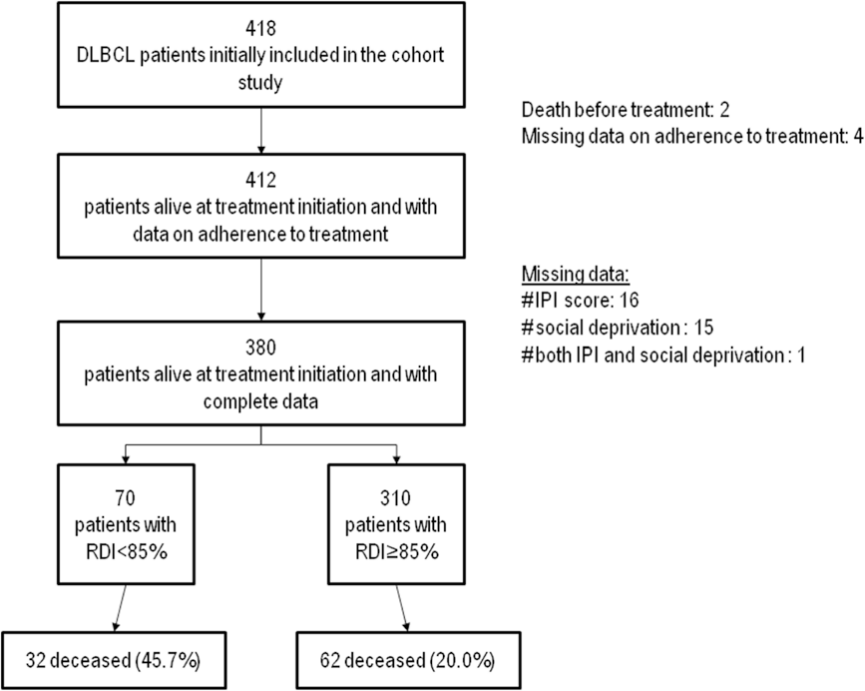
Flowchart.

Among the 418 patients initially included in this study, 2 deceased before starting treatment and 4 had no data regarding RDI. Poor adherence to treatment concerned 17.5% (72/412) of all treated patients with data on adherence to treatment. The baseline characteristics of patients features are presented in Tables 1, 2 and 3 for the clinical characteristics, socio-demographical profiles and the place of treatment. Among these patients, 16 patients had no IPI score. Fifteen patients presented an incomplete or incorrect home address which did not allow finding the corresponding IRIS or the EDI score, and one patient had no data for both IPI and EDI. The final sample used for multivariate models included 380 patients (91% of the total sample). During the follow-up, 94 patients died and 70 had a treatment adherence (RDI < 85%). The flowchart is presented in Figure 1.

**Table 1:** **Clinical characteristics of the 412 DLBCL patients with data on RDI included in the AMARE cohort study and comparisons between RDI groups**

		Total		RDI < 85% (n = 72)		RDI ≥ 85% (n = 340)		P value^a^
		n	%	n	%	n	%	
**Gender**	**Male**	222	53.9	34	47.2	188	55.3	0.212
	**Female**	190	46.1	38	52.8	152	44.7	
**Age**	**<59 y**	146	35.4	14	19.4	132	38.8	<0.001
	**59 - 73 y**	135	32.8	20	27.8	115	33.8	
	**>73 y**	131	31.8	38	52.8	93	27.4	
**Comorbidity**	**none**	161	39.1	22	30.6	139	40.9	0.103
	**at least 1**	251	60.9	50	69.4	201	59.1	
**Standard International prognostic index** **(sIPI)**	**very good**	56	13.6	5	6.9	51	15	<0.001
	**good**	217	52.7	29	40.3	188	55.3	
	**poor**	122	29.6	37	51.4	85	25	
	**missing**	17	4.1	1	1.4	16	4.7	
**LDH**	**normal**	197	47.8	27	37.5	170	50	0.054
	**elevated**	215	52.2	45	62.5	170	50	
**B signs**	**absence**	336	81.6	61	84.7	275	80.9	0.445
	**presence**	76	18.5	11	15.3	65	19.1	
**Ann Arbor Stage**	**I-II**	142	34.5	15	20.8	127	37.4	0.007
	**III-IV**	270	65.5	57	79.2	213	62.7	
**Performance status**	**PS = 0-1**	385	93.5	69	95.8	316	92.9	0.598 ^b^
	**PS = 2-4**	27	6.6	3	4.2	24	7.1	
**Regimens**	**R- CHOP 21 or R-CHVP**	223	54.1	28	38.9	195	57.4	<0.001 ^b^
	**R- CHOP 14**	34	8.3	10	13.9	24	7.1	
	**R- ACVBP**	45	10.9	3	4.2	42	12.4	
	**R-mini CHOP or R-mini CHVP**	100	24.3	30	41.7	70	20.6	
	**other**	10	2.4	1	1.4	9	2.7	

In bivariate analyses, p-values derived from the chi2 test ^a^ or the Fisher Exact test ^b^ when the expected frequencies were less than 5.

DLBCL: diffuse large B-cell lymphoma; RDI: relative dose intensity; LDH: lactate dehydrogenase.

**Table 2:** **Socio-demographic characteristics of the 412 DLBCL patients with data on RDI included in the AMARE cohort study and comparisons between RDI groups**

		Total		RDI < 85% (n = 72)		RDI ≥ 85% (n = 340)		P value^a^
		n	%	n	%	n	%	
**Occupational status**	**active**	123	29.9	22	30.6	101	29.7	0.861
	**inactive/retired**	268	65.1	46	63.9	222	65.3	
	**missing**	21	5.1	4	5.6	17	5	
**Cohabiting status**	**not alone**	253	61.4	41	56.9	212	62.4	0.271
	**alone**	125	30.3	26	36.1	99	29.1	
	**missing**	34	8.3	5	6.9	29	8.5	
**Socioeconomic status (quintile of EDI national scores)**	**1: highly favoured**	74	18.0	12	16.7	62	18.2	0.101
	**2: favoured**	72	17.5	20	27.8	52	15.3	
	**3: intermediate level**	96	23.3	11	15.3	85	25	
	**4: deprived**	89	21.6	17	23.6	72	21.2	
	**5: highly deprived**	65	15.8	11	15.3	54	15.9	
	**missing**	16	3.9	1	1.4	15	4.4	

In bivariate analyses, p-values derived from the chi2 test ^a^.

DLBCL: diffuse large B-cell lymphoma; RDI: relative dose intensity; EDI: European deprivation index.

**Table 3:** **Place of treatment of the 412 DLBCL patients with data on RDI included in the AMARE cohort study and comparisons between RDI groups**

		Total		RDI < 85% (n = 72)		RDI ≥ 85% (n = 340)		P value^a^
		n	%	n	%	n	%	
**Place of treatment**	**Private centres**	104	25.2	22	30.6	82	24.1	0.002
	**TUMC**	180	43.7	18	25	162	47.7	
	**Community hospitals**	128	31.1	32	44.4	96	28.2	

In bivariate analyses, p-values derived from the chi2 test ^a^.

DLBCL: diffuse large B-cell lymphoma; RDI: relative dose intensity; TUMC: Toulouse university medical centre.

The results of the bivariate analyses in Tables 1, 2 and 3 shown that RDI < 85% was associated with age, comorbidity, LDH, IPI, Ann Arbor Stage, type of treatment, socioeconomic status and place of treatment. Table 4 presents the results of the multivariate model studying the effects of clinical characteristics, socio-demographic profiles and place of treatment on the risk of having a poor RDI. Regarding clinical characteristics, poor RDI was favoured by advanced age, high risk IPI and treatment with R-CHOP 14. For socio-demographic characteristics, no socioeconomic gradient was found but we observed a protective effect of being in intermediate level compared to the highly favoured level. Finally, we found that being cared for in academic centres may protect against poor adherence to treatment.

**Table 4:** **Factors associated with receiving a relative dose-intensity lower than 85% - results of a multivariate logistic regression model (n = 380)**

		Odds ratios	p-value	[95% Confidence Interval]
**Gender**	**Male**	1		
	**Female**	1.32	0.361	[0.73; 2.37]
**Age** ^**a**^	**<59 y**	1		
	**59 - 73 y**	1.06	0.902	[0.41; 2.77]
	**>73 y**	4.42	0.019	[1.27; 15.35]
**Socioeconomic status** ^**b**^ **(quintile of EDI national scores)**	**1: highly favoured**	1		
	**2: favoured**	1.49	0.387	[0.60; 3.67]
	**3: intermediate level**	0.32	0.025	[0.12; 0.86]
	**4: deprived**	0.79	0.625	[0.31; 2.01]
	**5: highly deprived**	0.72	0.526	[0.26; 1.98]
**Comorbidity**	**none**	1		
	**at least 1**	1.13	0.704	[0.59; 2.18]
**Standard International prognostic index** ^**c**^ **(sIPI)**	**very good**	1		
	**good**	1.32	0.612	[0.45; 3.86]
	**poor**	4.60	0.008	[1.48; 14.30]
**Chemotherapy regimens** ^**d**^	**R- CHOP 21 or R-CHVP**	1		
	**R- CHOP 14**	7.65	0.001	[2.35; 24.92]
	**R- ACVBP**	1.30	0.737	[0.15; 6.06]
	**R-miniCHOP or R-mini CHVP**	0.66	0.429	[0.24; 1.87]
	**other**	0.09	0.046	[0.01; 0.96]
**Place of treatment** ^**e**^	**Private centres**	1		
	**TUMC**	0.23	0.003	[0.09; 0.60]
	**Community hospitals**	1.11	0.780	[0.55; 2.23]

Notes ^a^, ^b^, ^c^, ^d^, and ^e^ indicate the global p-value; ^a^: p = 0.027; ^b^: p = 0.025; ^c^: p < 0.001; ^d^: p = 0.002; ^e^: p = 0.002.

DLBCL: diffuse large B-cell lymphoma; RDI: relative dose intensity; EDI: European deprivation index; TUMC: Toulouse university medical centre.

**Figure 2:**
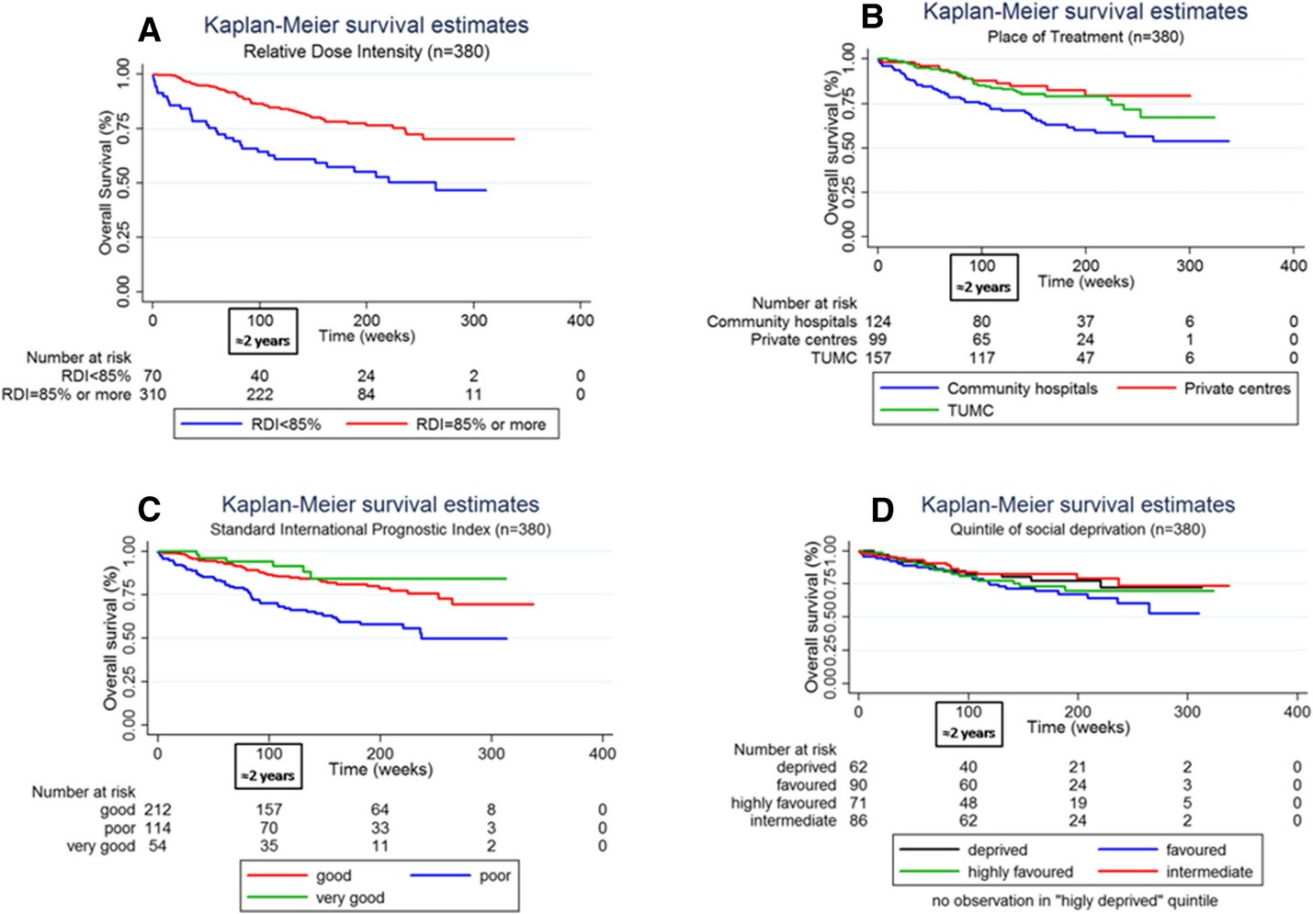
Kaplan-Meier survival estimates curves stratified by relative dose intensity **(A)**, place of treatment **(B)**, standard international prognostic index **(C)** and quintile of social deprivation **(D)**.

For survival analyses, the median follow-up was 994 days and the maximum length of follow-up was 2363 days. The year of diagnosis was not associated with overall survival (data not shown). As shown in the Kaplan-Meier’s curves plotted in Figure 2, poor RDI was associated with reduced overall survival (a reduction of about 25% at 24 month). The place of treatment seemed also influence overall survival with a reduced survival in community hospitals compared to private and academic centres. However, we found no socioeconomic gradient in overall survival. Analyses of Schoenfeld’s residuals showed a violation in the proportional hazard assumption for RDI (data not shown). Figure 2A suggests indeed that RDI < 85% more negatively influenced overall survival during the first 24-month period. That is why we introduced an interaction term between RDI and time in the Cox multivariate model noted as RDI*time in Table 5. Poor overall survival was associated with poor RDI. The significance of the RDI*time variable means that the negative effect on overall survival of having a RDI < 85% decreased with duration from the chemotherapy initiation. Complementary analyses showed that RDI < 85% reduced overall survival only during the first 24 month after treatment initiation (adjusted hazard ratio [95% confidence interval] = 3.23 [1.84; 5.69]). Table 5 shows no effect of the socioeconomic status on overall survival. Moreover, overall survival was higher for patients cared for in private hospitals compared to public academics or community centres (p-values = 0.068 and 0.075 respectively). Table 5 shows also poorer survival among patients with advanced age and poor IPI. Women had a better overall survival. No differences were found between chemotherapy regimens.

**Table 5:** **Factors associated with overall survival - results of a multivariate Cox regression model with the relative dose-intensity entered as a time dependent variable (n = 380)**

		Hazard ratio	p-value	[95% Confidence interval]
**Gender**	**Male**	1		
	**Female**	0.52	0.003	[0.34; 0.80]
**Age** ^**a**^	**<59 y**	1		
	**59 - 73 y**	1.77	0.153	[0.81; 3.88]
	**>73 y**	2.57	0.070	[0.92; 7.15]
**Socioeconomic status** ^**b**^ **(quintile of EDI national scores)**	**1: highly favoured**	1		
	**2: favoured**	0.91	0.783	[0.47; 1.78]
	**3: intermediate level**	1.46	0.242	[0.78; 2.73]
	**4: deprived**	0.61	0.171	[0.30; 1.24]
	**5: highly deprived**	0.74	0.429	[0.36; 1.55]
**Comorbidity**	**none**	1		
	**at least 1**	0.74	0.224	[0.46; 1.20]
**Standard International prognostic index** ^**c**^ **(sIPI)**	**very good**	1		
	**good**	1.30	0.559	[0.54; 3.15]
	**poor**	2.61	0.041	[1.04; 6.57]
**Chemotherapy regimens** ^**d**^	**R-CHOP21 or R-CHVP21**	1		
	**R- CHOP 14**	0.57	0.246	[0.22; 1.48]
	**R- ACVBP**	0.97	0.956	[0.32; 2.90]
	**R-mini CHOP or R-mini CHVP**	1.94	0.122	[0.84; 4.48]
	**other**	1.49	0.516	[0.45; 4.99]
**Relative dose-intensity (RDI)**	**RDI ≥85%**	1		
	**RDI <85%**	3.89	<0.001	[1.86; 8.14]
**Place of treatment** ^**e**^	**Private centres**	1		
	**TUMC**	1.88	0.068	[0.95; 3.71]
	**Community hospitals**	1.75	0.075	[0.95; 3.25]
**Time dependant variables**	RDI * Time	0.998	0.024	[0.997; 0.999]

Notes ^a^, ^b^, ^c^, ^d^, and ^e^ indicate the global p-value; ^a^: p = 0.176; ^b^: p = 0.083; ^c^: p = 0.007; ^d^: p = 0.338; ^e^: p = 0.133.

RDI * Time is the interaction term between RDI and time in the Cox multivariate model.

DLBCL: diffuse large B-cell lymphoma; RDI: relative dose intensity; EDI: European deprivation index; TUMC: Toulouse university medical centre.

## Discussion

In this population-based prospective cohort study, we found poor adherence, defined as RDI < 85%, in 17.5% of the treated patients (72/412). We showed that advanced age, poor IPI and treatment with R-CHOP 14 favoured RDI < 85%, as expected. Treatment in the academic centre TUMC was associated with RDI ≥ 85%. The results of our survival analyses designated poor adherence to treatment as strongly associated with poor overall survival independent of patients’ age, gender, socioeconomic status, comorbidity, IPI score, chemotherapy regimens and the place of treatment. We showed that patients treated in private centres were likely to have a better survival that those treated in public community hospitals and academic centre, after adjusting for confounders. Patients' socioeconomic status assessed by the level of social deprivation of their living area at the time of diagnosis had no effect on neither adherence to treatment nor overall survival.

In this study, we selected patients from the regional cancer network and we cannot generalise our results to the national level. At the regional level, we focused on the main centres covered by the network and thus we may have lost in representativeness. About 10% of the initial sample was excluded from our analyses because of missing data. In addition, the time period for including patients was almost five years. As a consequence, patients included at the end of the inclusion period may be more prone to be censored and thus they have less time to reach the event of interest than those included at the beginning of the period. Moreover, we had no data on what led to reduction in RDI and we could not know if it was a patient’s refusal or trepidation to receive treatment because of side effect, a physician’s decision in a case of a frail patient or a protocol-driven decision. However, this study was based on population data which should well reflect routine practice. This study deals with both medical and non-medical determinants of the treatment adherence and overall survival among patients treated for DLBCL in France. Data collection was prospective and about 90% of the total sample had complete data. Moreover, our models included patients’ socioeconomic status assessed by a European ecological index of social deprivation used as a proxy of the individual status.

Adherence to therapy has been established for years as a critical parameter for clinical benefit in medical oncology. This statement was established two decades ago for conventional chemotherapy in breast cancer [30,31] and lymphoma patients [32]. In the present study, we considered adherence to chemotherapy from an ecological point of view as we assume that adherence may be influence not only by characteristics of the individual patient, but also by factors within the patient's environment, or so-called system level factors. In an ecological model, patients' behaviour may be influenced by factors at the patient-level, micro- (provider and social support), meso- (health care organization), and macro (health policy) -levels [33]. In our study, about 17.5% of the total sample had less than 85% of the RDI. This relatively small proportion of patient with poor adherence to treatment may be explained as the use of G-CSF was widespread in our practices (data not shown), considering that prophylactic GCSF use is associated with increased RDI [29]. Our results suggest a strong effect of advanced age, treatment and poor IPI on RDI. These results are in agreement with the factors identified to be related to low RDI listed in Wildiers and Reiser’s review which encompasses increased age (>60 years), ECOG status, stage or IPI score and more occasionally, the type of treatment (ACVBP, CHOP14) or the use of G-CSF (secondary or primary prophylaxis) [17]. Our results have also pointed out a protector effect of being treated in the academic centre TUMC. Understanding the factors unique to this centre are key to revealing potential pathways though which RDI may be affected. A higher treatment adherence in TUMC may translate a higher experience of the medical team in dealing with side-effects and more complex case and feeling comfortable with maintaining the treatment despite these. In addition in this centre, DLBCL patients benefit from a telephone-based intervention by an oncology-certified nurse which consists of systematic calls to the patients twice a week during treatment and the collection of clinical and biological observations. The information is then forwarded to the oncologist, and corresponding interventions are performed [34]. As R-CHOP is administered through intra-venous route, it should not be influenced by patients’ attitude although the telephone-based intervention might improve the patient-physician relationship and patient’s positive appraisal of the treatment centre which have been pointed out as important factors in adherence to treatment [35]. However, it is possible that the telephone-based intervention set up in TUMC improved the management of side effects and secured the whole treatment, encouraging physicians to preserve dose-intensity. Moreover, we assume that this telephone-based intervention might improve physician adherence by increasing patients’ information and therapeutic education. This “physician non adherence” encompasses non adherence to recommendations, dose or temporal concession due to documented toxicity in agreement with recommendations, but also physician individual decision [36]. The latter had not been thoroughly investigated, essentially because it resides in the privacy of oncology practice. Indeed, it integrates various medical, psychological and social factors related to the patient (like the age) but also to the physician [37]. In the present study, the absence of data regarding what led to reduction in RDI limited our capability to interpret these results regarding adherence to treatment. Further studies are needed to disentangle which causes of RDI reduction may be attributable to the physician and to the patient. Such studies should not only look for clinical factors, classically identified as determinant of RDI [38], but also for non-medical characteristics of patients and their environment.

The impact of RDI on outcome in lymphomas treated with CHOP and related regimens, has been investigated before the introduction of rituximab [9-11,15,16]. Since the introduction of rituximab at the end of the 90s, some studies have supported the association between RDI and patient outcome but they were based on analyses of relatively small study samples [13,14]. To our knowledge, the present study is the first to explore the association between RDI and OS in the Rituximab era in a larger scale study sample while studying non-medical potential determinants of RDI, in particular the role of some socioeconomic factors and the place of treatment. In the present study based on a larger sample, our results suggest a strong association between poor adherence to treatment and the overall survival with an overall mortality almost four-times greater among patients with RDI < 85% than among those with RDI ≥ 85%. This association was lost after about two-years after the treatment initiation. This may reflect the fact that, for a patient newly treated for DLBCL, the risk of dying from a cause related either to his disease or the treatment diminishes with time since the treatment initiation due to the competition with the risk of dying from other causes unrelated to the disease over time. The results of a recent study published by Maurer et al. tended to support this observation as they found no difference in overall survival between DLBCL patients achieving 24 months of event-free survival from diagnosis and the age- and sex-matched general population [39]. The models we used in the present study were all adjusted for baseline IPI scores which lessened the risk of a reverse causation bias between in interpreting the relationship between RDI and overall survival. Indeed, a high IPI score may be considered as risk factor of pejorative disease evolution by including the stage of the disease and the presence of more than one extra nodal site. In the main analysis as well as in sensitivity analyses, the hazard ratio assessing the association between RDI and overall survival remained stable after adjusting for IPI and confounders suggesting no major confounding bias (data not shown). Additional information about the causes of dose concession and delay in treatment would have been informative but at present these data are not available.

A major concern of modern oncology lies in applying evidence-based medicine to routine medical practice in small scale private centres or community hospitals. In 2009, a study among lymphoma patients showed that treatment in rural community hospitals was associated with poorer overall survival than treatment in academic centres, whatever the geographical location and patients’ risk-profile with the exception of high-risk patient among whom urban academic centres was associated with the best outcome [22]. A more recent study among DLBCL patients pointed out the poorer overall survival of patients living in small or medium urban area compared to those living in rural or large urban areas [40]. In our study, we did not provide direct information regarding spatial disparities of patients’ outcomes as we focused on place of treatment that was academic centre, community hospitals or private centres. We showed that patients treated in private centres tended to have a better overall survival than those treated in public centres, academic or not (global p-value for the place of treatment variable, p = 0.133). This may reflect an unequal repartition of patients between the different types of healthcare centres which, in the private sector, may lead to an underrepresentation of high-risk-of-dying-patients. However, multivariate analyses adjusted for comorbidities and IPI showed no interaction between these variables and the care modalities. Another explanation may arise from the geographical distribution of the healthcare centres in the region corresponding roughly to academic centres in large urban areas, private centres in large and medium urban areas and the community health centres in small urban and rural areas. Further investigations based on complementary data for the characterisation of the spatial and structural environment of patients would be necessary to formally test these hypotheses. This is the purpose of an ongoing project.

Regarding the role of patients’ socioeconomic status, we found a protector effect of the intermediate socioeconomic level against poor treatment adherence. More data would be need concerning the place of residence or the occupation to help us in the interpretation of this result. Finally, we found no association between patients’ socioeconomic status assessed by the European ecological deprivation index (EDI) of the living area at diagnosis and overall survival in contrast with studies supporting social inequalities in survival and treatment of Non-Hodgkin’s lymphomas [19-21]. A possible explanation of the absence of socioeconomic gradient in overall survival may arise from the fact that the cohort was constituted by patients treated for DLBCL with the standard therapy. Indeed, the selection of such a population allows to observe patients only once they enter to the healthcare system but does not account for those who encountered difficulties in access to primary care which is a critical step in the healthcare trajectory of cancer patients [41,42]. In our study sample, we observed no association between patients’ IPI at diagnosis and their socioeconomic status suggesting that no social gradient in the distribution of this characteristic in our sample (data not shown). Another element which may explain the absence of effect of patients’ socioeconomic status is the way in which healthcare is organized in France. The policy of the regional cancer network dedicated to cancer patients, including haematological malignancies, dictates that all e-medical files are systemically screened by disease-specific boards constituted by university hospital staff members. Thus, our patients may have benefited from the expertise of the university hospital staff, independent of their socioeconomic status or their living areas. These results suggest that the French healthcare system is doing fairly well in absorbing the social inequalities in health among patients treated for DLBCL, that is once patients have overcome the barrier of primary access to care.

## Conclusions

This prospective study among patients treated for DLBCL with R-CHOP and R-CHOP like regimens in France yields information about the adherence to treatment and its association with overall survival in a “real life” setting. Our results suggest that poor adherence to treatment is strongly associated with overall survival with a risk of death almost four-time greater among patients with RDI < 85% compared with those with RDI ≥ 85%, principally during the first two-years after the initiation of the treatment. About 17.5% of the whole treated patients in this study received less than 85% of the planned treatment which was associated with advanced age and a high risk profile. Conversely, treatment in academic medical centres favoured a good adherence to treatment. As these centres have developed a telephone-based intervention by an oncology-certified nurse to monitor patients’ treatment, this warrants further research as a potential for the management of adverse effects. No effect of patients’ socioeconomic gradient was found on either adherence to treatment or overall survival.

## Abbreviations

DLBCL: Diffuse large B-cell lymphoma
NHL: Non-Hodgkin’s lymphomas
RDI: Relative dose-intensity
EDI: European ecological deprivation index
IPI: International prognostic index
